# The Effect of Lithospermic Acid, an Antioxidant, on Development of Diabetic Retinopathy in Spontaneously Obese Diabetic Rats

**DOI:** 10.1371/journal.pone.0098232

**Published:** 2014-06-06

**Authors:** Cheng Ji Jin, Sung Hoon Yu, Xiao-Mei Wang, Se Joon Woo, Hyo Jin Park, Hyun Chul Lee, Sung Hee Choi, Kyoung Min Kim, Jung Hee Kim, Kyong Soo Park, Hak Chul Jang, Soo Lim

**Affiliations:** 1 Department of Internal Medicine, Seoul National University College of Medicine and Seoul National University Bundang Hospital, Seongnam, Korea; 2 Department of Ophthalmology, Seoul National University College of Medicine and Seoul National University Bundang Hospital, Seongnam, Korea; 3 Department of Pathology, Seoul National University College of Medicine and Seoul National University Bundang Hospital, Seongnam, Korea; 4 Department of Endocrinology, The ZhongShan Hospital, DaLian University, Dalian, China; 5 Department of Internal Medicine, Hallym University Hangang Sacred Hospital, Seoul, Korea; 6 Department of Internal Medicine, Yonsei University College of Medicine, Seoul, Korea; 7 Department of Internal Medicine, Seoul National University College of Medicine, Seoul, Korea; Medical College of Wisconsin, United States of America

## Abstract

**Background:**

Lithospermic acid B (LAB), an active component isolated from *Salvia miltiorrhiza radix*, has been reported to have antioxidant effects. We examined the effects of LAB on the prevention of diabetic retinopathy in Otsuka Long-Evans Tokushima Fatty (OLETF) rats, an animal model of type 2 diabetes.

**Methods and Findings:**

LAB (10 or 20 mg/kg) or normal saline were given orally once daily to 24-week-old male OLETF rats for 52 weeks. At the end of treatment, fundoscopic findings, vascular endothelial growth factor (VEGF) expression in the eyeball, VEGF levels in the ocular fluid, and any structural abnormalities in the retina were assessed. Glucose metabolism, serum levels of high-sensitivity C-reactive protein (hsCRP), monocyte chemotactic protein-1 (MCP1), and tumor necrosis factor-alpha (TNFα) and urinary 8-hydroxy-2′-deoxyguanosine (8-OHdG) levels were also measured. Treatment with LAB prevented vascular leakage and basement membrane thickening in retinal capillaries in a dose-dependent manner. Insulin resistance and glucose intolerance were significantly improved by LAB treatment. The levels of serum hsCRP, MCP1, TNFα, and urinary 8-OHdG were lower in the LAB-treated OLETF rats than in the controls.

**Conclusions:**

Treatment with LAB had a preventive effect on the development of diabetic retinopathy in this animal model, probably because of its antioxidative effects and anti-inflammatory effects.

## Introduction

Diabetes mellitus has shown an exponential rise of late, causing serious economic, social and health repercussions. However, the causes of these problems and therapeutic options have yet not been fully uncovered. Among the side effects, diabetic retinopathy is the most common microvascular complication of individuals with both type 1 and type 2 diabetes, with nearly all patients with type 1 diabetes and more than 60% with type 2 diabetes being estimated to develop diabetic retinopathy 20****years after onset [1], [2]. Proliferative diabetic retinopathy, the advanced stage of diabetic retinopathy, is characterized by the extensive formation of new blood vessels in the retina and invasion towards the vitreous body, followed by vitreous hemorrhaging and tractional retinal detachment, leading to visual impairment and irreversible blindness [3], [4]. Although strict glycemic control has been shown to reduce the incidence of retinopathy considerably, it is not easily achieved or maintained. Its efficacy has also not been completely confirmed, given that 12% of patients with diabetes under intensive treatment develop retinopathy [1], [5].

While laser treatment at best permits preservation of visual acuity in cases of diabetic retinopathy, many agents such as fenofibrate, ruboxistaurin, renin-angiotensin system blockers, and peroxisome proliferator-activated receptor gamma agonists have been used in attempts to delay or prevent diabetic retinopathy [6]. A recent reanalysis of two major randomized controlled trials – the Fenofibrate Intervention and Event Lowering in Diabetes (FIELD) and the Action to Control Cardiovascular Risk in Diabetes (ACCORD) studies – revealed that fibrate has a robust beneficial effect on diabetic retinopathy [7]. Recently, a new approach to inhibit angiogenesis in cases of diabetic retinopathy has been studied. Several clinical trials indicate that vision can improve with repeated injections of bevacizumab [8], ranibizumab [9] and pegaptanib [10]. One study also showed that the intravitreal injection of bevacizumab inhibited VEGF expression in the retina and that bevacizumab-chitosan nanoparticles had a longer duration of action [11].

It is currently believed that retinopathy is an inflammatory process, because various studies have shown that the circulating leukocytes activated in diabetic patients play an important role in the increased adhesion of leukocytes, macrophages and vascular adhesion molecules, and in the process of vascular occlusion and retinal ischemia [12]–[14]. Oxidative stress in the retina is also fundamental for angiogenic stimulation, which is mediated by growth factors, including vascular endothelial growth factor (VEGF) [15], [16].

Today, antioxidants are known to be effective against microvascular complication such as diabetic nephropathy as well as macrovascular complications of diabetes such as myocardial infarction, atherosclerosis and stroke [17], [18]. However, its potential efficacy against diabetic retinopathy, a microvascular complication of diabetes, has not been adequately explored or proven.

*Salvia miltiorrhiza radix* is a traditional Chinese herbal medicine that has been used for many years in East Asia for the treatment of diabetic complications. Lithospermic acid B (LAB), a recently isolated component of *S. miltiorrhiza*, is known to have multiple pharmacological actions such as antihypertensive [19] and antioxidant effects [20]. Interestingly, a recent study has shown that LAB protects pancreas beta cells against cytokine-induced apoptosis [21].

Treatment with LAB showed protective effects against micro- and macrovascular complications of diabetes [22]–[24]. Moreover, treatment with LAB prevented both injury-induced neointimal formation *in vivo* and platelet-derived growth factor-induced vascular smooth muscle cell proliferation and migration *in vitro* [23]. LAB treatment prevented diabetic nephropathy by inhibiting reactive oxygen species generation, protein kinase C (PKC) activation, and transforming growth factor (TGF)-β1 and fibronectin expression [22]. Considering similar phenomena are involved in the pathogenesis of diabetic retinopathy [25], [26], LAB treatment might serve as a potential preventive therapeutic measure for diabetic retinopathy.

Reactive oxygen species have been reported to be associated with microvascular complications in patients with diabetes [27]. The DNA adduct 8-hydroxy-2′-deoxyguanosine (8-OHdG) is an indicator of oxidative stress damage [28]. A number of recent reports suggest that a low concentration of 8-OHdG following antioxidant therapy might reflect a reduced risk of diabetic complications [29], [30]. These findings raise the possibility that LAB treatment might reduce the risk of retinopathy and that reduced levels of oxidative stress markers such as 8-OHdG might be associated with such efficacy. Based on the assumption, we investigated the effects of LAB on the occurrence of retinopathy and aimed to identify the mechanisms involved using an animal model of spontaneous diabetes.

## Methods

### Animals

All animals were handled in compliance with the Guide for Experimental Animal Research of the Laboratory, Seoul National University Bundang Hospital. Seoul National University Hospital Ethics Committee for Animal Study approved this study (63-2010-020). Thirty five-week-old male Otsuka Long-Evans Tokushima Fatty (OLETF) rats, an obese animal model of insulin resistance, were donated by the Otsuka Pharmaceutical Co. (Tokushima, Japan). They were allowed to grow to 24****weeks of age, when obesity and insulin resistance develop. The OLETF rats were held in the Preclinical Laboratory of Seoul National University Bundang Hospital, South Korea, for the duration of the study.

We divided the rats into three groups (n = 10 each). Controls received 5****mL normal saline per day and the experimental rats were given 10****mg/kg or 20****mg/kg of LAB per day. Rats were administered with LAB or normal saline using an oral Zonde needle (Natsume, Tokyo, Japan) at 9–10 am for 52****weeks. All rats were fed a regular chow diet and had free access to water; they were maintained in plastic cages in an air-conditioned room at 22±2°C and 55±10% humidity.

### Isolation and purification of lithospermate B

Lithospermate B was isolated from 80% MeOH extract of *Salviae miltiorrhizae* Radix, and subsequently purified by normal silica gel column chromatography with polar-eluent (isopropyl alcohol/H_2_O/ethyl acetate  = 3/1/3) to give a 1.01% yield. Acidification of magnesium lithospermate B with 0.5****N-HCl afforded lithospermic acid B at an 84% yield [31].

### Fundus photography and fluorescein angiography

Fundus photography on both eyes of each animal was performed by an experienced technician using a 45° fundus camera (Retinal Camera CR6-45NM; Canon Inc., Tokyo, Japan) at week 0, 12, 24, 36, and 52 of the dosing phase. Fundus fluorescein angiography was performed on both eyes at 52****weeks. All retinal photographs and fluorescein angiography images were independently reviewed twice by one ophthalmologist (SJW), and the retinal vascular caliber was measured in each blood vessel 1****mm distant from the optic disc and averaged for each eye.

### Eyeball extraction and histological analysis

Eyeballs were enucleated from rats (both control and LAB groups) after euthanasia with a lethal dose of pentobarbital and fixed in 10% neutral buffered formalin solution. They were embedded in paraffin wax. Sections (4 µm thickness) perpendicular to the optic disc were stained with hematoxylin and eosin (H&E) for light microscopy. Digital images were obtained using a high-resolution digital camera system (C3040-AD6, Olympus, Tokyo, Japan) linked to a microscope (CH30, Olympus, Tokyo, Japan) and desktop computer. For all eyes, retinal images were obtained at the same distance (1500 µm) from the optic nerve.

### Immunohistochemistry

Sections were deparaffinized in xylene, rehydrated in a graded ethanol series, and stained using a BenchMark XT automated immunostainer (Ventana, Tucson, AZ, USA). Immunohistochemistry was performed using an anti-VEGF antibody (Clone C1, 1:500 dilution, Santa Cruz Biotechnology, Santa Cruz, CA, USA). Sections were washed with phosphate buffered saline (PBS) and incubated with a secondary antibody (anti-mouse IgG, istostain-Plus Bulk Kits; Zymed, San Francisco, CA, USA), prepared in PBS containing 1% horse serum, 1% bovine serum albumin and 0.3% Triton X-100, for 1****h at room temperature. Slides were washed with PBS.

### VEGF levels in ocular fluid

Immediately after the rats were euthanized, the VEGF concentration was measured in ocular fluid–a mixture of the aqueous humor and vitreous fluid–by puncturing the ocular globe using disposable 1****mL syringes and 30 gauge needles. Samples were stored at −70°C until assayed. The VEGF concentration was measured with a rat VEGF-specific polyclonal antibody using a commercial enzyme-linked immunosorbent assay (ELISA) (Quantikine ELISA, R&D Systems, Minneapolis, MN, USA). The detection limit of the method was 31.2–2000 pg/mL, based on a standard curve from the manufacturers. All measurements were carried out in duplicate. The coefficients of variation in intra- and interassay variations were 7.2% (79.4±5.7 pg/mL) and 9.4% (87.6±8.2 pg/mL), respectively.

### Assessment of retinal capillary basement membrane thickness

Electron microscopy was performed to determine the retinal basement membrane thickness. The eyes were enucleated, opened at the equator, fixed in 3.5% glutaraldehyde in 0.1****M cacodylate buffer (pH****7.4) for 1****h, and then postfixed in 2% osmium tetroxide. They were then dehydrated in ethanol series, and embedded in epoxy resin. Semithin sections (1 µm) were stained with toluidine blue for orientation and identification of the capillaries. Thin sections (60****nm) were cut with a diamond knife, placed on 300-mesh copper grids, and stained with uranyl acetate and lead citrate. The sections were viewed and photographed using a JEOL JEM-100SX transmission electron microscope (JEOL, Tokyo, Japan). Each micrograph was analyzed using a commercial image analysis program (Image Pro 4.5, Media Cybernetics, Inc., Rockville, MD, USA). Basement membrane thickness was measured on four distinct capillaries for each eye and five measurements were taken per capillary with 10 independent measurements from each eye. One researcher (SJW) examined all eyes in the study and was blinded to the treatment groups.

### Western blot analysis for quantification of VEGF in the retina

Tissue samples were homogenized in lysis buffer, and the protein concentration was determined using a protein assay kit (Pierce Biotechnology, Rockford, IL, USA). Extracted retinal tissues were lysed in RIPA buffer (25****mM Tris-HCl pH****7.6, 150****mM NaCl, 1% NP-40, 1% sodium deoxycholate, 0.1% SDS), and the proteins (10****mg/sample) were immediately heated at 100°C for 3****min. Total cell lysates were subjected to sodium dodecyl sulfate–polyacrylamide gel electrophoresis on gels containing 15% (wt/vol) acrylamide with each lane receiving 20 µg of the sample. After electrophoresis, proteins were transferred onto a nitrocellulose membrane for 2****h. The membrane was blocked by treatment with 5% skim milk in Tris-buffered saline (TBS) supplemented with 0.1% Tween-20 (TBST) for 1****h. It was then incubated with a primary polyclonal antibody (rabbit polyclonal to VEGF, Abcam Inc., Cambridge, MA, USA) at a final dilution of 1:1000, overnight in TBST. After three washes in TBST, the membrane was incubated with peroxidase-conjugated secondary antibody (final dilution, 1:3000) in TBS for 1****h and subsequently washed. An enhanced chemiluminescence reaction agent was reacted with the membrane for 3****min. Band density was quantified using a densitometer (Bio Image analyzer, CN-115, ETX-20MX, Marne-la-Vallée, France).

### Serum levels of LAB in study animals

Levels of LAB were measured using a liquid chromatography-tandem mass spectrometry (LC-MS/MS) system from plasma samples collected at the end of the study. The standard curve was linear (r^2^  = 0.994) over the concentration range of 1.0–1,000 µg/mL. The intra- and interassay coefficients of variation were 6.2% and 2.7%, respectively. The mean serum concentrations of LAB were 12.3±2.4 and 17.1±3.1 µg/mL in the rats treated with 10 and 20****mg/kg of LAB, respectively. LAB was not detected in the control rats.

### Glucose metabolism, and inflammatory and prothrombotic markers

Possible relevant factors affecting the degree of diabetic retinopathy, such as glucose homeostasis, cytokines, and inflammatory status were evaluated. For glucose homeostasis, postprandial glucose concentrations were measured 2****h after refeeding on every other week from weeks 26 to 50. An intraperitoneal glucose tolerance test (IPGTT) was done at baseline and after 24 and 52****weeks of LAB treatment. After the 12****h fasting glucose concentration had been measured, each animal was injected intraperitoneally with 1.5 g/kg of a 50****M glucose solution. Blood samples (about 10 µL) were collected from an incision in the tail at 30, 60, 90 and 120****min after the glucose load. Plasma glucose concentration was measured using reagent strips read in a glucose meter (YSI 2300-STAT, Yellow Springs, OH, USA). Insulin was measured in duplicate using a commercial liquid phase radioimmunoassay kit (Linco Research, St Louis, MO, USA). The homeostasis model assessment of the insulin resistance (HOMA-IR) was calculated using fasting insulin and glucose levels. In addition, the area under the curve for glucose (AUC_glucose_) was calculated using the trapezoid rule for glucose data from 0 to 120****min. High sensitivity C-reactive protein (hsCRP) was measured using ELISA kits developed by BD Biosciences Pharmingen (Heidelberg, Germany). Monocyte chemotactic protein-1 (MCP1), tumor necrosis factor-alpha (TNFα), and plasminogen activator inhibitor-1 (PAI1) activity were also measured using a multiplex kit (RADPK-81K, Linco, Billerica, MA, USA).

### Measurement of 8-hydroxy-2′-deoxyguanosine (8-OHdG), an oxidative stress marker, in urine

After 52****weeks of treatment of LAB or control, urine samples were collected directly from the urinary bladder with a 10****mL syringe on the day of euthanasia after fasting the rats overnight. The oxidative DNA damage marker, 8-OHdG was measured in the urine samples using an established high performance liquid chromatographic method based on anion exchange chromatography, fraction collection, and electrochemical detection [28]. The measurement of 8-OHdG (in micrograms) was normalized to urine creatinine (in grams).

### Statistical analysis

Results are reported as the mean ± standard error (SE). Mean values were compared for the LAB-treated groups and control group by analysis of variance (ANOVA) with Tukey's b *post hoc* test, and *P* values <0.05 were considered statistically significant. Analysis was done using SPSS for Windows (version 17.0; IBM Corp., Armonk, NY, USA).

## Results

### Fundus images and microvasculature

**Figure 1:**
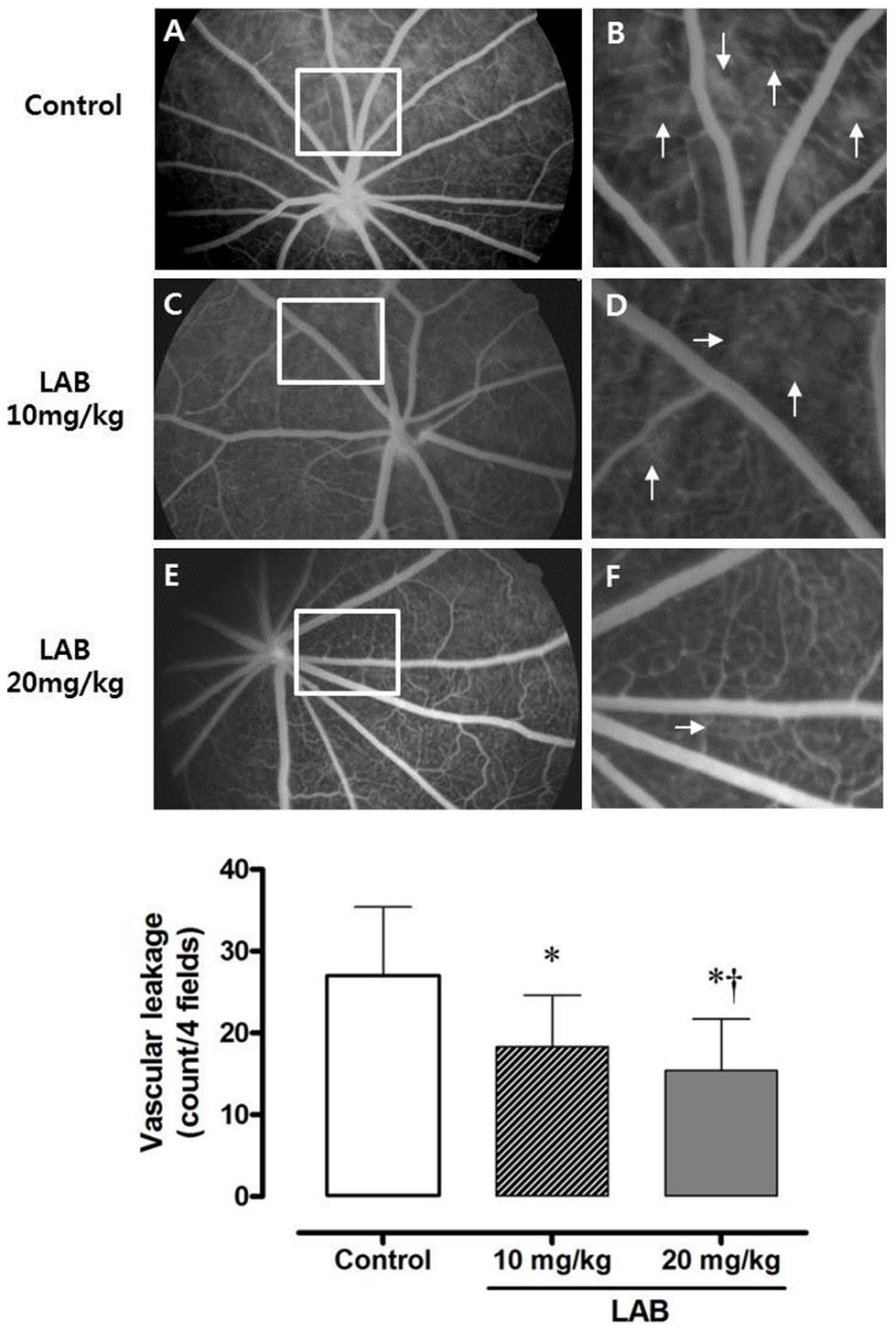
Vascular leakage in fluorescein angiography in study animals. *A, B*: Control group. *C, D*: 10****mg/kg LAB group. *E, F*: 20****mg/kg LAB group. Arrows indicate vascular leakage. *G:* Comparison of vascular leakage among three groups. *P<0.05 vs. control group, †*P*<0.05 vs. 10****mg/kg LAB-treated group.

Images of the fundus from the control group showed dilated retinal vasculature compared with the LAB-treated groups, but this was not significant: 67±6.9 µm in controls; 58.3±7.8 µm in the 10****mg/kg LAB group, 53.2±8.8 µm in the 20****mg/kg LAB group (**Figure S1 in File S1**). Fluorescein angiography allowed more precise quantitative analysis of diabetes-induced microvascular dysfunction. At the end of treatment, the diabetic rats (control group) showed multiple diffuse leakages from retinal capillaries in the late phase of angiography (**Fig. 1** ***A, B***). The LAB-treated retinal angiograms showed a significantly lesser degree of vascular leakage compared with untreated control rats (**Fig. 1** ***C, D*** for the 10****mg/kg LAB group and **Fig. 1** ***E, F*** for the 20****mg/kg LAB group; Comparison among groups was shown in **Fig. 1** ***G***).

### Retinal H&E staining and retinal thickness

**Figure 2:**
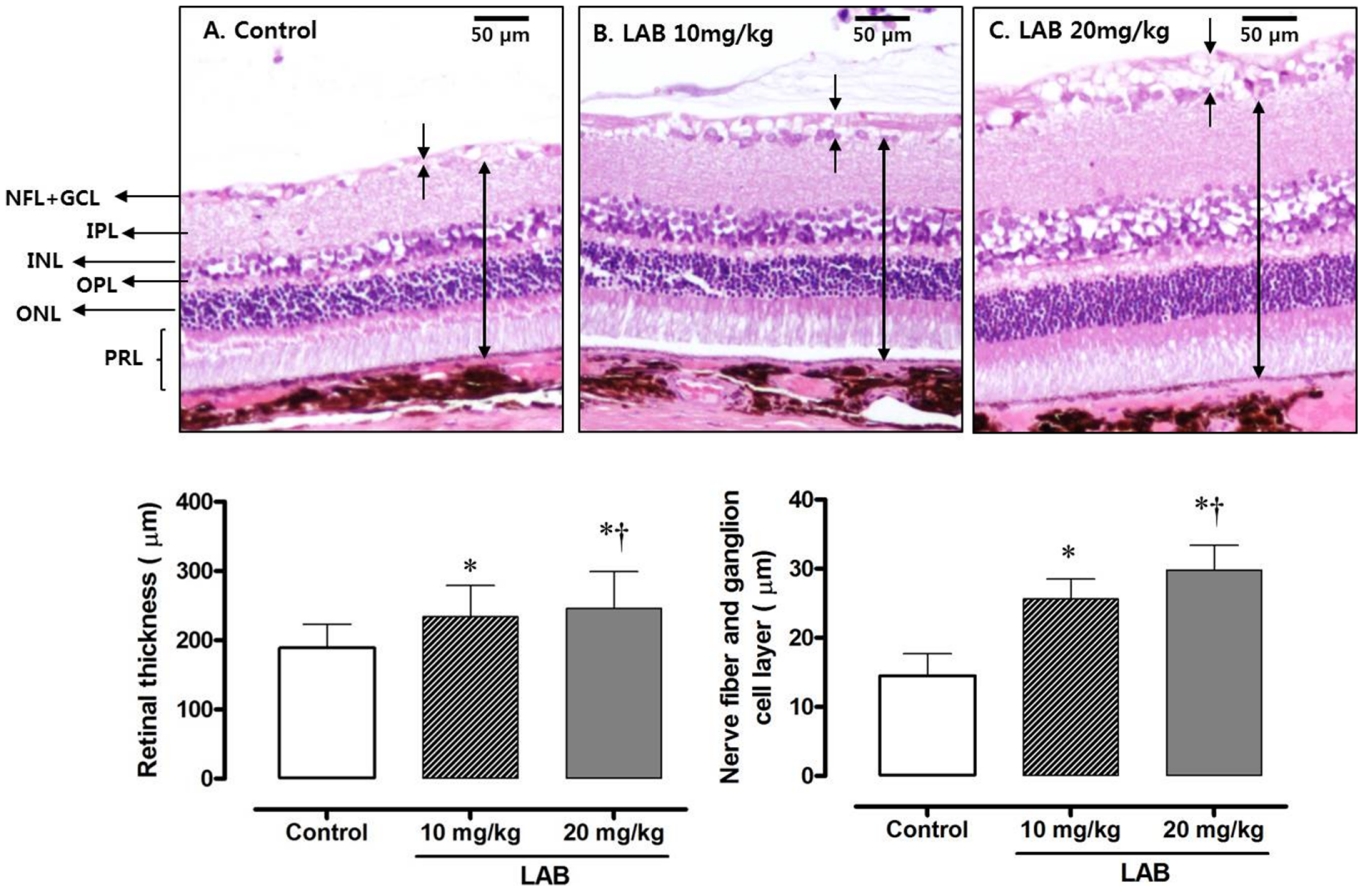
Histology of the rat retina using H&E staining. *A*: Control retina. *B*: 10****mg/kg LAB-treated retina. *C*: 20****mg/kg LAB-treated retina. Key: NFL, nerve fiber layer, GCL; ganglion cell layer; IPL, inner plexiform layer; INL, inner nuclear layer; OPL, outer plexiform layer; ONL, outer nuclear layer; PRL, photoreceptor layer.

After 52****weeks of treatment of LAB, microscopic examination of H&E-stained retinas showed no gross morphological abnormalities in study animals (**Fig. 2**). However, the retinas of the control group were slightly but significantly thinner than LAB-treated groups. Further, LAB-treated rats showed thicker nerve fiber and ganglion cell layers than did control rats, in a dose-dependent manner.

### Vessels in the retina and in the surrounding connective tissues

**Figure 3:**
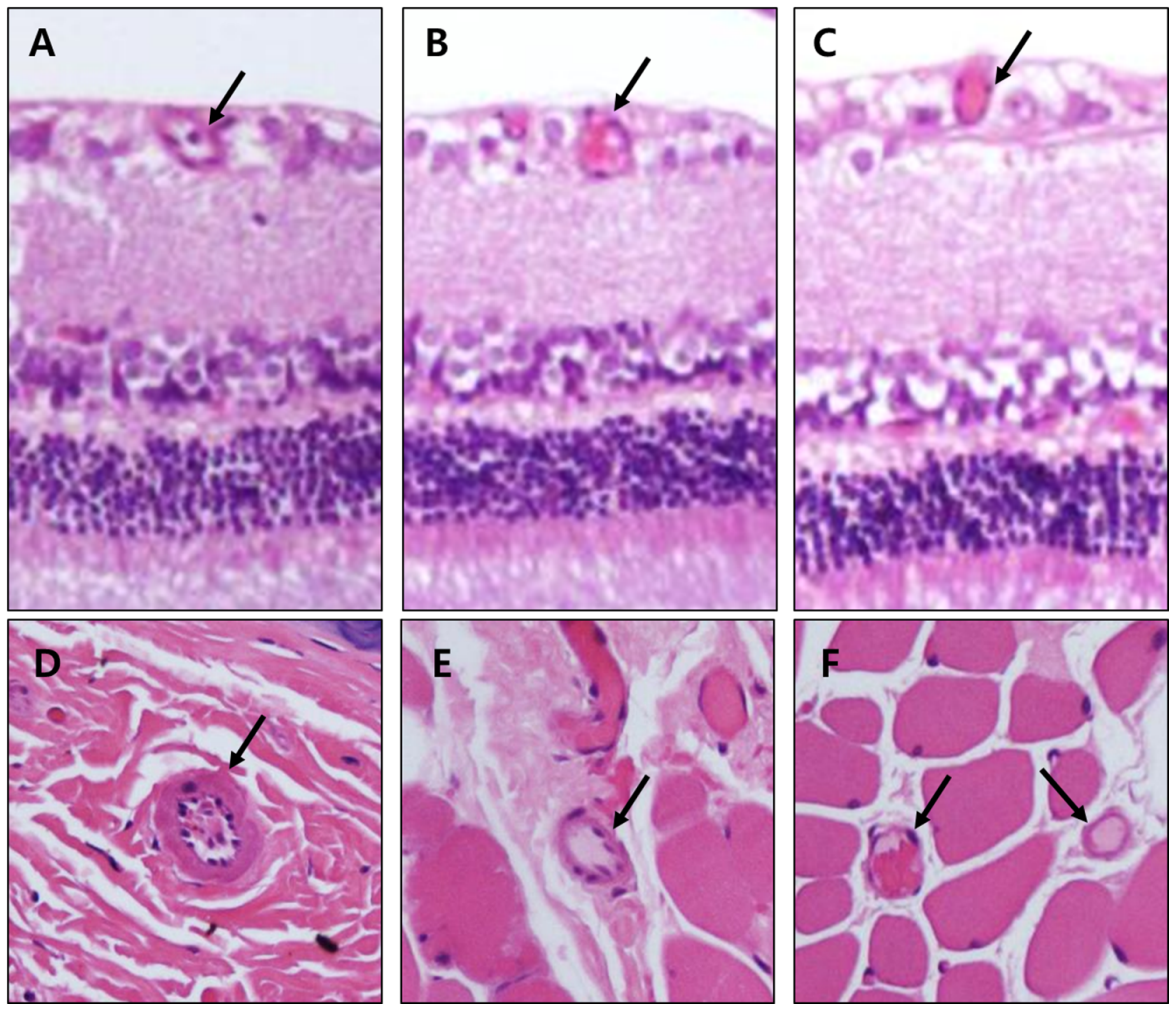
Capillaries in rat retinas and in surrounding connective tissues (× 200). *A, D*: Control. *B, E*: 10****mg/kg LAB treatment. *C, F*: 20****m/kg LAB treatment. Arrows indicate capillaries.

As shown in **Fig. 3**, retinal capillaries in the control rats showed atherosclerotic changes including wall thickening and dilatation. Such atherosclerotic changes were not apparent in the LAB-treated rats.

### Western blot results for VEGF expression in the retina

**Figure 4:**
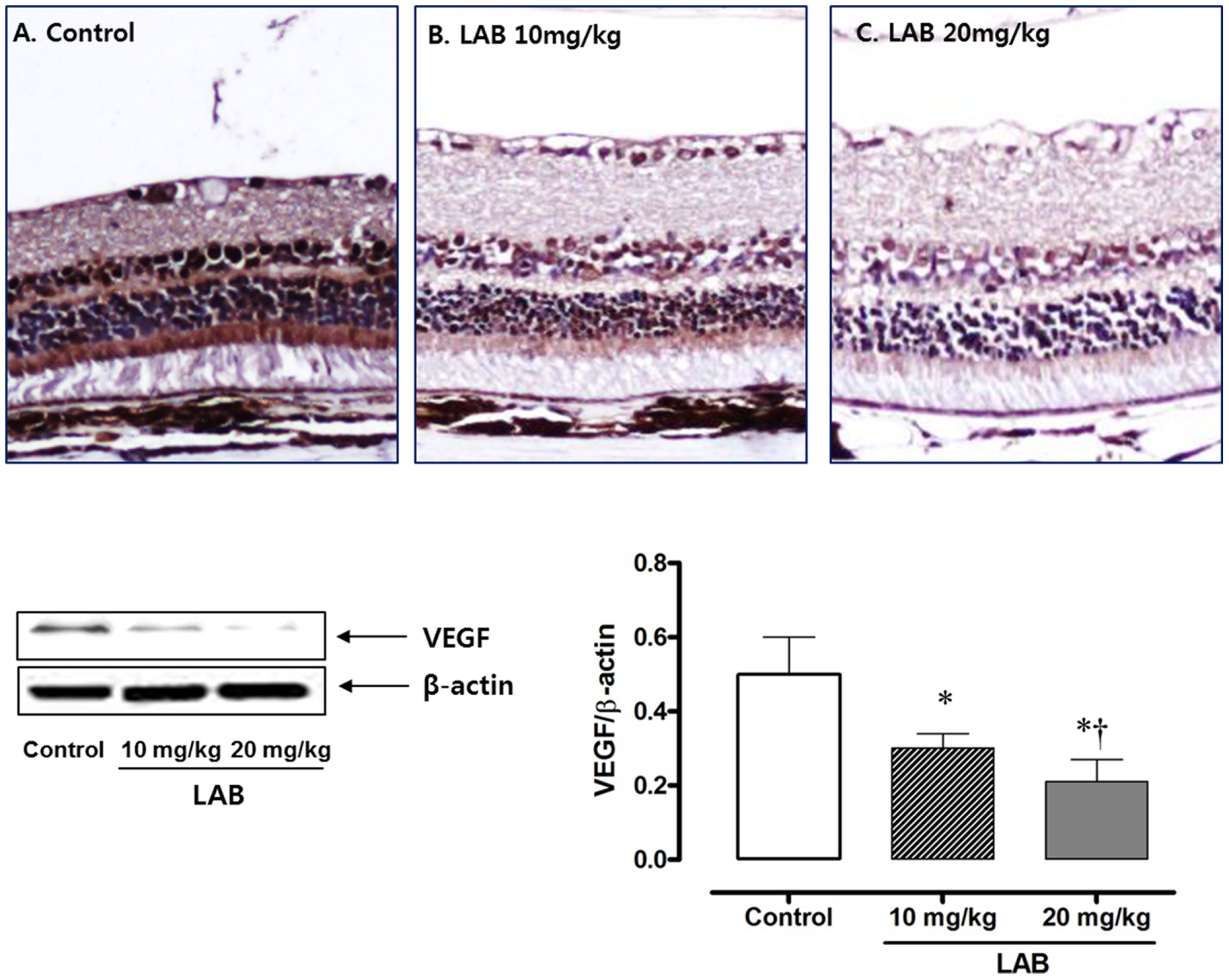
Immunohistochemistry for VEGF and western blot analysis of VEGF expression in the retina. *A*: Control group. *B*: 10****mg/kg LAB-treated group. *C*: 20****mg/kg LAB-treated group. *P<0.05 vs. control group, †*P*<0.05 vs. 10****mg/kg LAB-treated group.

Immunohistochemical staining showed greater VEGF expression in the control diabetic rats than in the LAB-treated rats (**Fig. 4**). In the western blot analysis, the levels of VEGF were decreased in the LAB-treated groups compared with the control group (*P*<0.05). The high dose (20****mg/kg) LAB-treated group showed lower VEGF expression than did the low dose group (*P*<0.05).

### VEGF levels in ocular fluid

**Figure 5:**
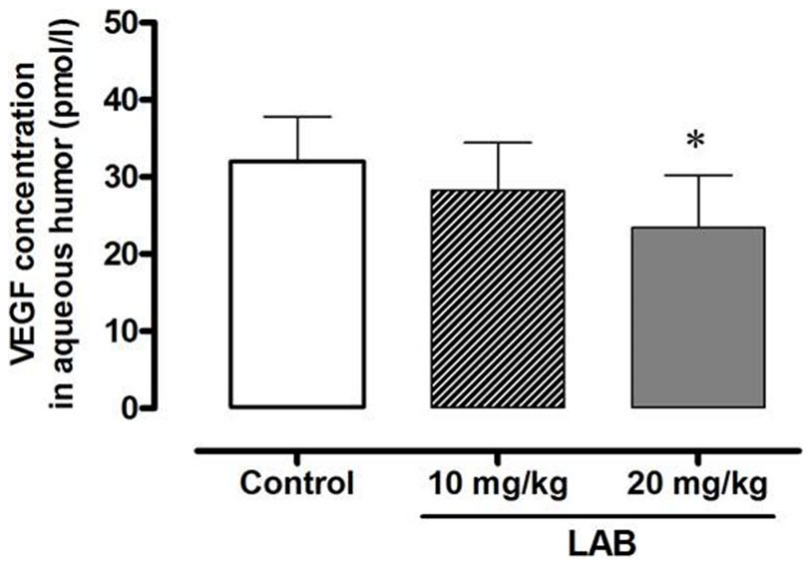
VEGF concentrations in ocular fluid among three groups. Each column represents mean ± SE (n = 5–6). *P<0.05 vs. control group.

Ocular fluid was obtained from five control, six 10****mg/kg LAB-treated, and five 20****mg/kg LAB-treated rats. The concentration of VEGF in the high dose treated group was significantly lower than in control rats **(** **Fig. 5** **).**

### Retinal capillary basement membrane thickness

**Figure 6:**
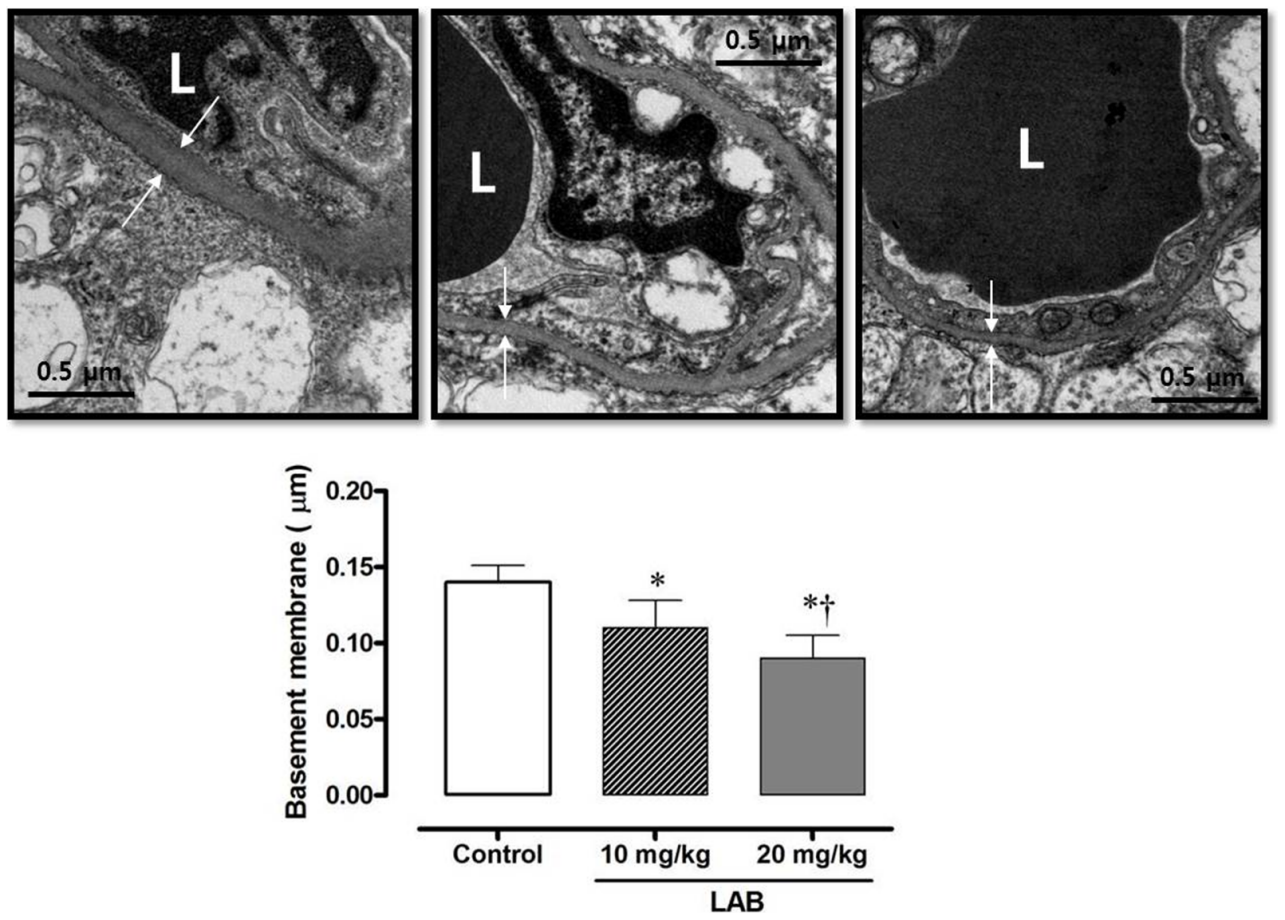
Retinal capillary basement membrane thickness assessed by electron microscopy. *P<0.05 vs. control group, †*P*<0.05 vs. 10****mg/kg LAB group.

Retinal capillary basement membrane thickness was assessed by electron microscopy. For each animal, a minimum of 40 separate measurements of the basement membrane thickness was obtained. A representative micrograph of the basement membrane from each of the three groups is shown in **Fig. 6**. The basement membrane in control diabetic rats was thicker than in the LAB-treated rats and there was a dose-dependent relationship for the measures: 0.14±0.01 µm in the control group, 0.11±0.2 µm in the 10****mg/kg LAB group, and 0.09±0.02 µm in the 20****mg/kg LAB group, *P*<0.05 among groups).

### Effects of LAB treatment on glucose metabolism and biochemical parameters including markers associated with inflammatory processes, prothrombotic status, and oxidative stress

Postprandial 2****h glucose concentrations from weeks 24 to 50 (**Figure S2 in File S1**) revealed that control rats had higher glucose concentrations at weeks 46 and 48 than did the LAB-treated rats. There was no difference in glucose excursion for the IPGTTs performed at week 24 among the three groups (**Figure S3 in File S1**). After 52****weeks of LAB treatment, the 90****min and 2****h postload glucose concentrations and the AUC_glucose_ during the IPGTT decreased significantly compared with that of the controls (*P*<0.05; **Table 1** and **Figure S4 in File S1**).

**Table 1:** Weight, biochemical parameters, insulin resistance index and inflammatory markers in an obese rat model of type 2 diabetes after 52****weeks of treatment with lithospermic acid B or with saline as control.

	Control (n = 10)		10 mg/kg LAB (n = 10)		20 mg/kg LAB (n = 10)		*P* value with *post hoc*†
	Mean	SD	Mean	SD	Mean	SD	
Weight, g	609.1	34.8	613.7	35.5	621.3	45.2	
Fasting glucose, mg/dL	128.6	19.6	121.2	9.9	118.8	12.8	
30 m postload glucose, mg/dL	405.2	13.3	385.5	16.1	371.2	16.1	
60 m postload glucose, mg/dL	459.4	20.8	443.6	17.0	435.1	22.6	
90 m postload glucose, mg/dL	372.7	34.0	354.6	24.7	311.5	21.2	<0.05^A,B^
2 h postload glucose, mg/dL	236.3	56.2	201.8	46.1	189.4	29.8	<0.05^A,B^
AUC_glucose_	709.9	53.0	672.6	42.9	636.0	40.6	<0.05^A,B^
Fasting insulin, pg/mL	156.2	78.2	145.1	69.3	128.7	59.2	
HOMA-IR	44.2	19.2	40.1	16.1	37.8	11.1	<0.05^B^
Total cholesterol, mg/dL	82.6	15.3	81.9	12.9	77.6	9.9	
Triglycerides, mg/dL	78.1	24.4	63.4	21.8	60.2	15.8	
HDL-cholesterol, mg/dL	21.4	5.6	22.8	6.1	28.1	2.9	
LDL-cholesterol, mg/dL	49.0	13.1	42.6	11.9	43.1	7.1	
HsCRP, mg/L	0.16	0.03	0.12	0.01	0.10	0.01	<0.05^A,B^
TNFα, pg/mL	9.9	2.9	7.6	3.8	5.9	4.1	<0.05^B^
MCP1, pg/mL	156.2	57.2	123.9	43.4	107.2	35.2	<0.05^A,B^
PAI1, pg/mL	512.5	182.5	422.8	103.9	456.8	128.4	
8-OHdG/creatinine, mg/g	8.2	2.6	6.2	1.8	5.1	0.9	<0.05^A,B^

Key: AUC_glucose_, Area under the curve of glucose; HDL, high-density lipoprotein; HsCRP, high-sensitivity C-reactive protein; HOMA-IR, homeostasis model assessment of insulin resistance; LDL, low-density lipoprotein; MCP1, monocyte chemotactic protein-1; PAI1, plasminogen activator inhibitor-1; TNFα, tumor necrosis factor-alpha; 8-OHdG, 8-hydroxy-2′-deoxyguanosine. †ANOVA with *post hoc* test (Tukey's b) was used. A, B and C superscripts indicate significant differences between pairs of groups: A, Control vs. 10 mg/kg LAB group; B, Control vs. 10 mg/kg LAB group; C, 10 mg/kg LAB vs. 20 mg/kg LAB groups; *P*<0.05 for all cases.

A tendency towards improved insulin resistance was observed, as indicated by a reduction in the HOMA-IR. Treatment with LAB significantly decreased the serum levels of hsCRP and MCP1, well-known inflammatory markers. The levels of TNFα, also involved in inflammatory processes, also tended to decrease after LAB treatment, although the difference between the control and the 10****mg/kg LAB group was not significant. LAB treatment also reduced the level of 8-OHdG, an oxidative stress marker, in a dose-dependent manner (**Table 1**).

## Discussion

In this OLETF rat model of type 2 diabetes, treatments with LAB (10 and 20****mg/kg) for 52****weeks alleviated the development of diabetic retinopathy. Systemic LAB treatment prevented vascular leaking, reduced the expression and concentration of VEGF, and attenuated the disruption of the neurovascular units in the retina. The antioxidative and anti-inflammatory properties of LAB seem to account for this protective role. A significant improvement in postload glucose excursion with LAB treatment can be considered to contribute to this beneficial effect on the development of retinopathy.

Diabetic retinopathy is one of the biggest causes of blindness worldwide. Diabetic retinal capillary obstruction and resultant ischemic damage to the retinal neurons is irreversible, thus treatments to prevent the progression of diabetic retinopathy in the early stage should play a critical role in salvaging vision among patients with diabetes mellitus. In this sense, LAB has the potential to prevent the development and progression of diabetic retinopathy and related visual impairment.

The pathogenic mechanism in the development of diabetic retinopathy is complicated and has not been fully elucidated [32]. High serum and tissue levels of VEGF are the main culprit in the initiation and progression of retinal changes in patients with diabetes mellitus [33]–[35]. Oxidative stress and low grade inflammation as well as glycemic dysregulation increases the production of VEGF, which induces neovascularization, vascular leakage, and macular edema in the retina [34], [36]. Indeed, VEGF is known as a vascular permeability factor, based on its ability to induce microvascular leakage [16], [35]. In addition, VEGF plays an angiogenic and mitogenic role in vascular endothelial cells [37].

The mechanism of LAB in ameliorating the hyperglycemia-induced retinal vascular changes in OLETF rats seems to be associated with decreased VEGF expression in retina, as evidenced by reduced VEGF concentration in the retina and ocular fluid. PKC expression is also associated with VEGF-induced intraocular vascular permeability increases and neovascularization [38], [39]. Activation of PKC isoforms is responsible for pathologies in patients with diabetic retinopathy by modulating Na^+^-K^+^-ATPase [40]. Moreover, treatment with LAB inhibits PKC activation and reduces TGF-β1 and fibronectin levels in renal mesangial cells [22]. Considering the interrelationship between VEGF and PKC and their contribution to diabetic retinopathy, LAB might be a possible therapeutic option for the treatment of diabetic retinopathy *per se* or as an adjunct to anti-VEGF agents [41].

In our study, total retinal thickness and the combined nerve fiber and ganglion cell layers were maintained in the LAB-treated rats compared with the control diabetic rats. This finding is consistent with previous studies showing neurodegeneration and microangiopathic changes involved in diabetic retinopathy [42], [43]. In the milieu of diabetes mellitus, impaired insulin receptor signaling and loss of neurotrophic signals deteriorate cell survival and cell–cell interactions at synapses [44]. This can lead to an increase in apoptosis in the neural retina, and cause a breakdown of the blood–retinal barrier. Loss of this barrier integrity might also lead to compositional changes of the extracellular fluid in the retina, resulting in neuronal cell loss, especially in the ganglion cell layer. Atherosclerotic changes including wall thickening and dilatation were apparent in the retinal capillaries of control rats whereas treatment with LAB mitigated these changes. Collectively, it is conceivable that LAB maintains retinal thickness by attenuating the disruption of the neurovascular units of the retina.

Compared with the control diabetic rats, the basement membranes of retinal vessels were also maintained in the LAB-treated rats. Thickening of such basement membranes is a maladaptive response to impairment of glucose homeostasis and activation of various pathways associated with diabetic microvascular complications such as advanced glycation and increased PKC expression [32]. In a hyperglycemic milieu, synthesis of the basement membrane increases resulting in a thickened basement membrane caused by the accumulation of extracellular matrix [45]. Increased levels of VEGF, a growth factor and TNFα, an inflammatory cytokine contribute to thickening of the basement membrane [46].

Oxidative stress is also involved in the pathogenesis of diabetic retinopathy via activating angiogenesis and glial dysregulation [33]. In the present study, LAB treatment lowered urine 8-OHdG levels compared with control. This is a major marker of free radical-induced oxidative damage, and has therefore been used widely as an oxidative stress marker [47]. Taken together, the antioxidative and VEGF-reducing properties of LAB might be linked to neuroprotection and to maintenance of the integrity of the retinal basement membrane.

In this study, the LAB treatment reduced circulating MCP1 concentrations in a dose-dependent manner. MCP1 is a strong activator of monocytes and macrophages, suggesting that it is involved in the pathogenesis of diabetic retinopathy [48]. We also found that circulating levels of hsCRP and TNFα were significantly lower in the LAB-treated rats than in the untreated diabetic controls. In a large cohort study, the prevalence of retinopathy was positively associated with CRP levels in patients with diabetes [49]. Another study showed that patients with proliferative diabetic retinopathy had significantly higher serum TNFα levels compared with those with a milder form of retinopathy [50]. Moreover, the serum and vitreous humor levels of the soluble TNF receptor were elevated in patients with diabetic retinopathy [51]. Impairment of the blood–retinal barrier also leads to macrophage migration into the retina and accumulation of inflammatory mediators in the vitreous cavity, and vice versa. These findings suggest a potential role for LAB treatment in reducing oxidative stress and inflammatory responses in the pathogenesis of diabetic retinopathy.

In this study, we used OLETF rats, a rat model of obese type 2 diabetes with insulin resistance. Many investigators have used these rats as a model of diabetic retinopathy [43], [52], [53]. Thus, ganglion cell death was increased significantly in retinas from 35-week-old OLETF rats, and Akt phosphorylation was decreased compared with retinas from control LETO rats [53]. A recent study has shown that retinal neurodegeneration occurs in OLETF rats aged 36****weeks [43]. In another study with OLETF rats at the age of 40****weeks, VEGF expression extended into the outer plexiform layer, but expression was limited to the ganglion cell layer in control rats [52]. Thus, many studies have proven that alterations in retinal ultrastructure in OLETF rats are similar to those seen in human diabetic retinopathy, suggesting that the OLETF rat might be a useful animal model for the study of ocular complications in humans [54].

Although anti-VEGF therapy has been introduced for treating diabetic retinopathy, several limitations exist such as the need for intraocular injections, no clear rationale of use for preventive purposes, and the potential for aggravation upon discontinuing treatment, leading to a justification for the investigation of other therapies. A few studies suggest potential role of antioxidants in the prevention of diabetic retinopathy in human. L-carnitine, which facilitates the transport of long chain fatty acids into mitochondria, improves insulin sensitivity in insulin-resistant patients, suggesting a possible beneficial effect on the progression of diabetic retinopathy [55]. Recently, a preliminary human study suggested that α-lipoic acid treatment improved the electroretinogram results in patients with diabetic retinopathy [56]. However, the treatment period was only 4****weeks so the study could not reveal long-term results.

The present study had some limitations. First, the results of animal study cannot be extrapolated directly to humans with the same disease, although many studies have proven that the OLETF rat is a useful animal model for the study of human diabetic retinopathy. Second, we could not measure relevant biomarkers in ocular fluid because of its very small volume. Third, VEGF levels in ocular fluid were measured in limited numbers of animals because of difficulty in obtaining optimal volumes for the measurement.

In conclusion, our data demonstrate that LAB, an antioxidant, had preventive effects on the development of diabetic retinopathy in this obese type 2 diabetes rat model. These effects were probably mediated by its antioxidative effects and by reductions in inflammatory processes. Thus, LAB treatment might protect against the microvascular complications of diabetes, but further clinical studies are required to confirm this.

## Supporting Information

Checklist S1 (DOC)

File S1 **Supporting Figures.** **Figure a,** Fundoscopic findings in 76-week-old OLETF rats after 52****weeks of treatment with LAB. *A*: Control group. *B*: 10****mg/kg LAB group. *C*: 20****mg/kg LAB group. **Figure b,** Postprandial 2****h glucose concentrations from weeks 26 to 50 in the LAB-treated and control rats. **Figure c,** Glucose concentrations from the intraperitoneal glucose tolerance test (IPGTT) at week 24 in the LAB-treated and control rats. **Figure d,** Glucose concentrations from the intraperitoneal glucose tolerance test (IPGTT) at week 52 in the LAB-treated and control rats.
(DOCX)
