# Supplementary material for: The Effect of Lithospermic Acid, an Antioxidant, on Development of Diabetic Retinopathy in Spontaneously Obese Diabetic Rats
Source: PLoS One. 2014 Jun 6;9(6):e98232. doi: 10.1371/journal.pone.0098232 (PMC4048190; doi:10.1371/journal.pone.0098232)
Supplement: File S1 — Supporting Figures. Figure a, Fundoscopic findings in 76-week-old OLETF rats after 52weeks of treatment with LAB. A: Control group. B: 10mg/kg LAB group. C: 20mg/kg LAB group. Figure b, Postprandial 2h glucose concentrations from weeks 26 to 50 in the LAB-treated and control rats. Figure c, Glucose concentrations from the intraperitoneal glucose tolerance test (IPGTT) at week 24 in the LAB-treated and control rats. Figure d, Glucose concentrations from the intraperitoneal glucose tolerance test (IPGTT) at week 52 in the LAB-treated and control rats. (DOCX) [file pone.0098232.s002.docx]

**File S1: Supporting Figures.**


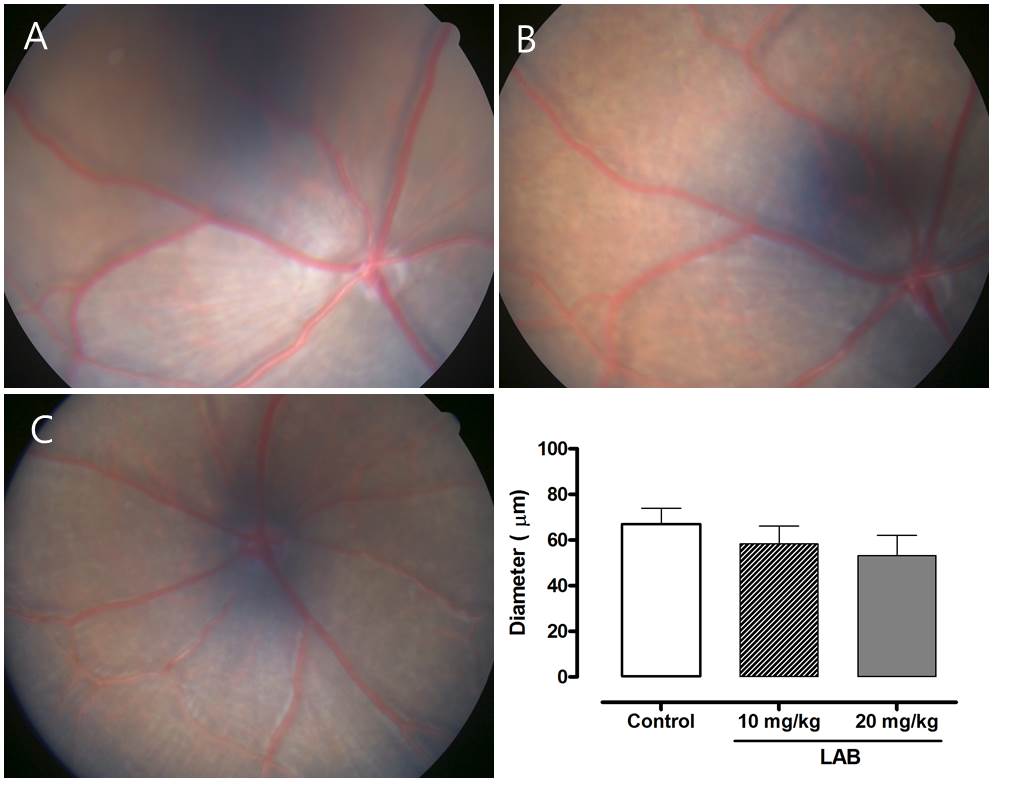


**Fig. a.** Fundoscopic findings in 76-week-old OLETF rats after 52 weeks of treatment with LAB. *A*: Control group. *B*: 10 mg/kg LAB group. *C*: 20 mg/kg LAB group.


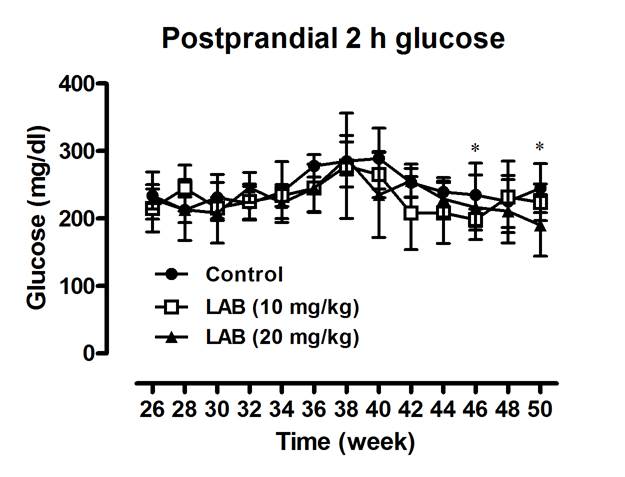


**Fig. b.** Postprandial 2 h glucose concentrations from weeks 26 to 50 in the LAB-treated and control rats


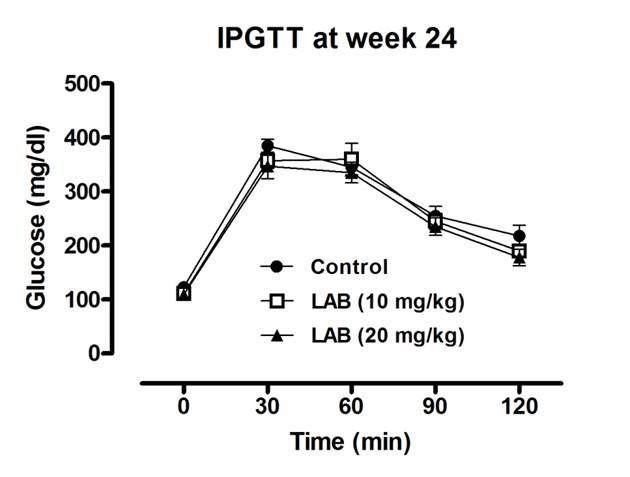


**Fig. c.** Glucose concentrations from the intraperitoneal glucose tolerance test (IPGTT) at week 24 in the LAB-treated and control rats


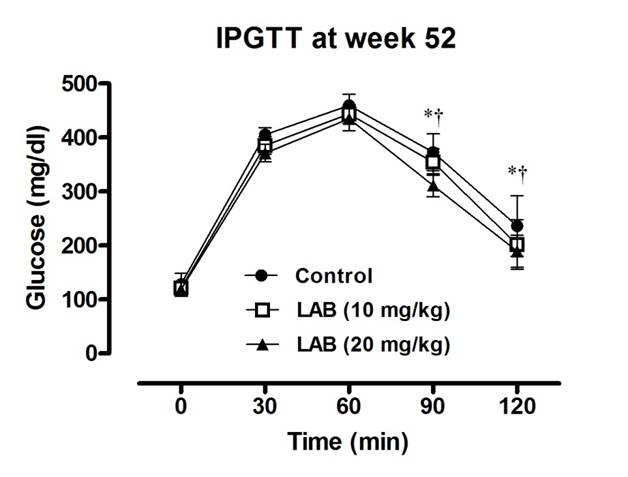


**Fig. d.** Glucose concentrations from the intraperitoneal glucose tolerance test (IPGTT) at week 52 in the LAB-treated and control rats
